# A Time-Domain Hybrid Analysis Method for Detecting and Quantifying T-Wave Alternans

**DOI:** 10.1155/2014/502981

**Published:** 2014-04-03

**Authors:** Xiangkui Wan, Kanghui Yan, Linlin Zhang, Yanjun Zeng

**Affiliations:** ^1^School of Electrical and Electronic Engineering, Hubei University of Technology, Wuhan 430068, China; ^2^School of Information Engineering, Guangdong University of Technology, Guangzhou 510006, China; ^3^Biomedical Engineering Center, Beijing University of Technology, Beijing 100022, China

## Abstract

T-wave alternans (TWA) in surface electrocardiograph (ECG) signals has been recognized as a marker of cardiac electrical instability and is hypothesized to be associated with increased risk for ventricular arrhythmias among patients. A novel time-domain TWA hybrid analysis method (HAM) utilizing the correlation method and least squares regression technique is described in this paper. Simulated ECGs containing artificial TWA (cases of absence of TWA and presence of stationary or time-varying or phase-reversal TWA) under different baseline wanderings are used to test the method, and the results show that HAM has a better ability of quantifying TWA amplitude compared with the correlation method (CM) and adapting match filter method (AMFM). The HAM is subsequently used to analyze the clinical ECGs, and results produced by the HAM have, in general, demonstrated consistency with those produced by the CM and the AMFM, while the quantifying TWA amplitudes by the HAM are universally higher than those by the other two methods.

## 1. Introduction

The T-wave alternans (TWA) has been considered as one of the most promising markers of sudden cardiac death (SCD) over the past 10 years. TWA is a phenomenon appearing in the surface electrocardiograph (ECG) as a consistent fluctuation in the repolarization morphology on an “every-other-beat” basis (2 : 1 behavior). This fluctuation refers to a beat-to-beat variability in the amplitude, morphology, and/or polarity of the T-wave. Numerous clinical studies have demonstrated that TWA is associated with ventricular arrhythmias. Nowadays TWA has been considered an independent predictor of cardiac arrhythmias.

Several signal processing methods have been proposed to detect and estimate TWA in the ECG on a single-lead or multilead basis [1–8]. And a comprehensive and systematic discussion of methods for TWA detection and analysis is reported in [9]. Most widely used TWA detection methods work in two different domains: time and frequency.

The disadvantage of the frequency based methods is that they treat the alternans signal as a stationary wave with the constant amplitude and phase, which is not true in general. They cannot detect nonstationary characteristics of the signal.

The time-domain methods can detect the TWA of nonstationary ECG signal in short time, and they have also been used on Holter data. The correlation method (CM) [6, 7], as a well-known time-domain method, performs well under different conditions, but it is sensitive to noise, especially to baseline wandering. In the presence of baseline oscillations at TWA frequency, a strong overestimation of TWA mean amplitude, and even TWA detection from TWA-free ECG tracings, is produced by the CM. And in the presence of higher frequency baseline fluctuations, the CM is not able to identify TWA [10]. An adapting match filter method (AMFM) was proposed by the same authors of the CM to overcome the CM limitations [11]. The AMFM yielded a significant improvement in the algorithm-based identification of duration and amplitude of TWA from ECG tracings with frequency of baseline oscillations both lower and higher than that of TWA. Nevertheless, in the presence of baseline fluctuations at the TWA frequency, it produced erroneous TWA detection from ECG tracing with no TWA and even strong overestimation of TWA amplitude, when present.

Based on above background, we propose a hybrid approach for the TWA detection, which is based on the correlation method and the least squares regression technique. The study aims to develop a novel TWA detector to overcome the CM limitations, which can detect and measure transient TWA with more accuracy in the time domain, even in the presence of higher frequency baseline fluctuations.

The rest of the paper is organized as follows. In Section 2, we present a novel method of TWA detection; simulated cases and clinical cases are also prepared. Then, in Section 3, we report the results of its validation on the simulation database and clinical database and compare the results to that of the CM and the AMFM. Next, in Section 4, we give the discussion. Finally, we summarize the conclusions of this work in Section 5.

## 2. Material and Methods

### 2.1. The Hybrid Analysis Method (HAM) Using Correlation Method and Least Squares Regression Technique

The hybrid analysis method consists of three different blocks: preprocessing, TWA detection, and TWA evaluation. The whole TWA analysis process is described as follows.

#### 2.1.1. Data Preprocess

Before detecting TWA, the clinical ECG used here are required to be submitted to a preliminary preprocessing stage. This consists of various steps, which are baseline wandering suppression, QRS complex detection, and segmentation of the T-wave.

(i) Baseline wandering suppression: this is performed using a cubic spline interpolation technique [12].
(ii) QRS complex detection: it is determined using a wavelet-based algorithm [13].
(iii) T-wave segmentation: it is done by selecting intervals of 300 ms, beginning at a distance from the QRS fiducial point dependent on the *RR* interval. The interval onset for the *i*th beat, *b*_*i*_, is given by the expression
  (1) $$\begin{array}{c} b_{i}=40+1.3RR^{1/2}\;\left(\text{ms}\right). \end{array}$$
(iv) T-wave alignment: after T-wave segmentation, 128 consecutive T-waves present in the ECG are used to compute the median T-wave (*T*_*m*_, which has each sample point given by the median value of the corresponding sample points of the 128 available T-waves), which is used as a template. Synchronization of the *i*th T-wave is performed according to a recursive procedure that keeps the segmented T-wave window length constant but varies its position ±30 ms from the original position, with a time increment of one sample point. For each position of the T-wave window, the windowed *i*th T-wave is cross-correlated against the template. Optimal alignment occurs when maximum correlation is reached.

#### 2.1.2. Qualitative Detection of TWA

After data preprocessing, TWA is detected by looking for an alternating trend in the T-wave morphology quantified by a correlation index. To this aim, an alternans correlation index (ACI) is computed to measure morphological changes of each of the consecutive *T*_*i*_ waves in comparison to *T*_*m*_ [12], which is as shown as follows:

(2) $$\begin{array}{c} {\text{ACI}}_{i}=\frac{\sum_{j=1}^{N}T_{i}\left(j\right)T_{m}\left(j\right)}{\sum_{j=1}^{N}{\left[T_{m}\left(j\right)\right]}^{2}}\;i=1,2,\ldots ,128, \end{array}$$

where *T*_*m*_ is the median T-wave computed using 128 T-waves available in each ECG tracing. *N* is the number of samples in each T-wave.

ACI_*i*_ is defined as the ratio of the maximum value of the cross-correlation function of *T*_*i*_ and *T*_*m*_ over the maximum value of the autocorrelation function of *T*_*m*_. *T*_*i*_ is classified as alternating.

**Figure 1:**
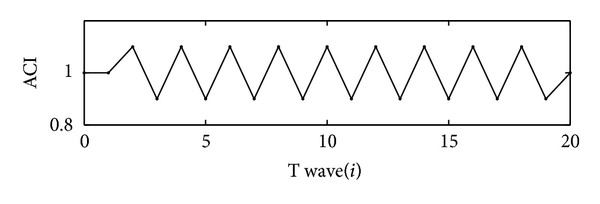
An example of the presence of TWA.

The presence of TWA is considered when the value of ACI strictly oscillates (not necessary around one) in the case of monophasic TWA for at least 7 consecutive beats.  Figure 1 shows an example of alternating values of ACI_*i*_, indicating the presence of TWA.

To limit false detections caused by noise, a local threshold criterion, with Th_ACI_ equal to 0.06 [6], is considered, such that ACI values alternations have to exceed 0.12 for at least seven consecutive beats to be detected as TWA.

#### 2.1.3. Quantitative Estimation of TWA

The odd and even beats of the above detected consecutive beats are labeled as *A* and *B*, respectively. The odd T-waves are obtained from *A* series and the even T-waves are obtained from *B* series. The odd T-waves constitute a matrix:

(3) TAm×n=(TA0,TA1,…,TAn)=[TA0,0TA0,1⋯TA0,nTA  1,0  TA1,1⋯TA1,n⋯⋯⋯⋯TAm,0TAm,1⋯TAm,n],

where *T*_*A*_*m*,*n*__ is the *n*th point of the *m*th odd T-wave. Analogously the even T-wave matrix *T*_*B*_*m*,*n*__can be constituted.

The amplitude corrections of odd and even T-waves are performed using the first-degree polynomial as shown below:

(4) $$\begin{array}{c} f\left(i\right)=aT_{i,k}+b, \end{array}$$

where *T*_*i*,*k*_ is the *i*th row and *k*th column point of odd (or even) T-wave matrix. And the coefficients *a*, *b* are estimated by the linear least squares fitting process.

Each column vector *T*_*A*_*k*__of *T*_*A*_*m*×*n*__ is divided into 7-point epochs, and (4) is recursively applied to each epoch throughout the entire *T*_*A*_*k*__. Denote *θ*_*i*_ as the *i*th deviation point of *T*_*A*_*k*__ from the fitting line:

(5) $$\begin{array}{c} \theta_{i}=\left|T_{i,k}-f\left(i\right)\right|. \end{array}$$

Then the mean deviation value of *T*_*A*_*k*__ can be expressed as follows:

(6) $$\begin{array}{c} \overset{-}{\theta }=\frac{\sum_{i=0}^{m}\theta_{i}}{m}. \end{array}$$

If $\max(\theta_{i})\geq 3\times \overset{-}{\theta }$, then the *θ*_*i*_ is considered to be corrected and replaced by the *i*th column mean value $\bar{T}(k)$ (as shown in (7)) of odd (or even) T-wave matrix. Consider

(7) $$\begin{array}{c} \overset{-}{T}\left(k\right)=\frac{\sum_{i=0}^{m}T_{i,k}}{m}. \end{array}$$

And the amplitude correction of the entire *T*_*A*_*k*__ is recalculated, until the $\max(\theta_{i})\leq 3\times \overset{-}{\theta }$ or max⁡(*θ*_*i*_)≺2 *μ*V.

**Figure 2:**
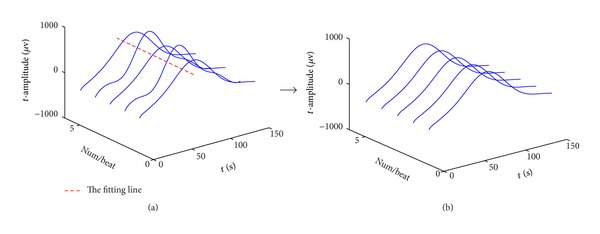
A specific example of amplitude correction of odd T-wave matrix.

A specific example of amplitude correction of odd T-wave matrix using the linear fitting function is shown in Figure 2. Figure 2(a) represents the uncorrected T-waves and Figure 2(b) represents the corrected T-waves.

The corrected matrixes for odd and even T-waves are known as $\bar{T_{A}}$ and $\bar{T_{B}}$, respectively. Measure TWA_*k*_ as the maximum absolute value of the difference between $\bar{T_{A_{k}}}$ and $\bar{T_{B_{k}}}$:

(8) $$\begin{array}{c} \text{TWA}\left(k\right)=\text{ma}{\text{x}}_{i=T_{\text{onset}}}^{i=T_{\text{offset}}}\left|\bar{T_{A_{k}}}\left(i\right)-\bar{T_{B_{k}}}\left(i\right)\right|, \end{array}$$

where TWA(*k*) denotes the *k*th local TWA (i.e., relative to a single odd (or even) beat), *k* = 1,2,…, *m*.

The TWA of the analyzed consecutive ECG segment (segment TWA) is measured as the mean value of measured local TWAs:

(9) $$\begin{array}{c} \text{TW}{\text{A}}_{\text{seg}}=\frac{\sum_{k=1}^{m}\text{TWA}\left(k\right)}{m}. \end{array}$$

And the global TWA (i.e., relative to the entire ECG tracing analyzed) is measured as the mean value of segment TWAs:

(10) $$\begin{array}{c} \text{TWA}=\frac{\sum_{i=1}^{l}\text{TW}{\text{A}}_{\text{seg}}\left(i\right)}{l}. \end{array}$$

**Figure 3:**
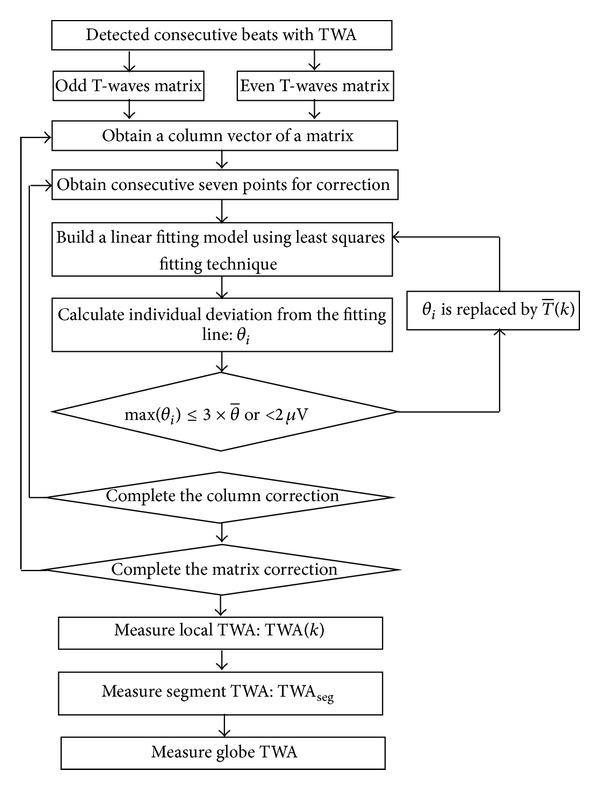
The block diagram of T-waves amplitude correction and TWA estimation.

The above process can be described as the block diagram (Figure 3).

### 2.2. Simulated Cases

There is no generally accepted TWA-measuring criterion to be used as a gold-standard. Therefore, a simulation approach was used in the present study in different controlled cases.

A realistic, clean simulated ECG was obtained as a K-fold repetition of a single beat extracted from a real ECG [14]. This guarantees that all the T-waves of the simulated ECG are identical, so no TWA can be present in the original signal. In particular, we used a 0.7 s beat sampled at 500 samples per second. The length of each simulated ECG tracing was assumed to count 128 consecutive heart beats. Our choice relies on the fact that 128 consecutive beats were originally used for SM applications, although some time-domain methods (e.g., modified moving average) use shorter ECGs [15]. A constant *RR* interval of 0.7 s was assumed, so that TWA fundamental frequency was 0.71 Hz (i.e., 1/(0.7 × 2 s) or 0.5 cycles per beat). TWA was simulated by varying T-wave amplitude (10, 50, and 100 *μ*V) in a time window of 160 ms centered around the T-wave apex.

Four different sets of ECG simulation were considered, respectively, reproducing the cases relative to the absence of TWA, the presence of stationary TWA, the presence of time-varying TWA, and the phase-reversal TWA, which are described below.

#### 2.2.1. Case 1: Simulated ECG Tracing with No TWA

**Figure 4:**
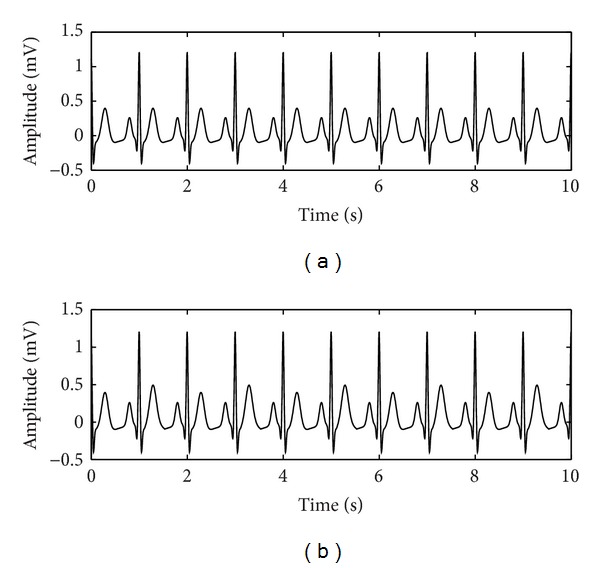
An example of simulated ECG tracings with/without TWA. (a) Simulated ECG tracings not affected by TWA (N_TWA); (b) simulated ECG tracings with stationary 50 *μ*V TWA (S_TWA50).

The simulated ECG tracing with no TWA (N_TWA) is assumed not to be affected by any kind of noise. This simulated signal is thought to test the ability of recognizing the absence of TWA, which is represented in Figure 4(a).

#### 2.2.2. Case 2: Simulated ECG Tracings with Stationary TWA

The simulated ECG tracings with stationary TWA (S_TWA) are designed to test the ability of quantifying TWA amplitude in the presence of stationary alternating T-wave profiles. Three kinds of simulated ECG tracings were considered, namely, a tracing with a 10 *μ*V TWA (S_TWA10), a tracing with a 50 *μ*V TWA (S_TWA50), and a tracing with a 100 *μ*V TWA (S_TWA100). An example of a tracing with a 50 *μ*V TWA is represented in Figure 3(b).

#### 2.2.3. Case 3: Simulated ECG Tracings with Time-Varying TWA

**Figure 5:**
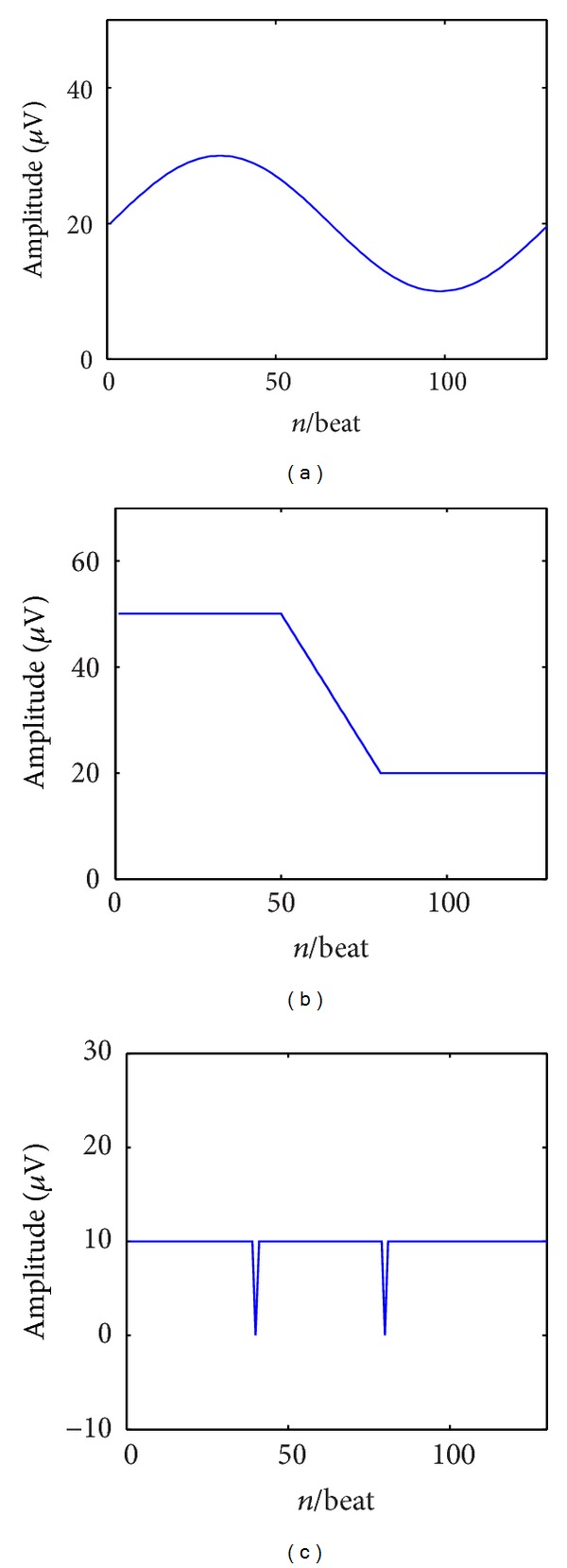
The different cases of nonstationary TWA. (a) Sinusoidal trend of TWA amplitude signals; (b) cascaded TWA amplitude signals; (c) phase-reversal TWA.

**Figure 6:**
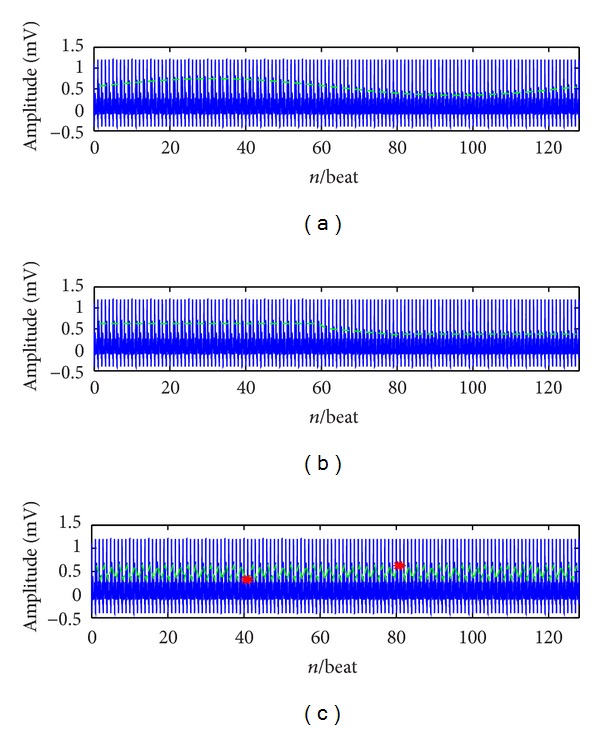
The simulated ECG tracings with different nonstationary TWA. (a) TV TWA1; (b) TV_TWA2; (c) PR_TWA.

ECG with visible TWA clearly shows the nonstationary nature of this phenomenon, whose variability often shows on-off or cyclic trends. Evaluation of dynamic aspects of TWA is important in clinics since transient TWA has been observed during acute ischemia [16]. To test the ability of the HAM in detecting nonstationary TWA, two simulated ECG tracings were considered, each one incorporating a specific beat-to-beat varying (and then, time-varying) *A*(*n*) sequence. Sinusoidal *A*(*n*) sequences, with 128 beats period were affecting the first (TV_TWA1) ECG tracing, while An *A*(*n*) varying from 50 *μ*V to 20 *μ*V, following a smoothed (24 beats transition) step pattern, was affecting the second ECG tracing (cascaded TWA, TV_TWA2). The two simulated tracings were characterized by a uniform profile of TWA, which are represented in Figures 5(a) and 5(b). The examples of TV_TWA1 and TV_TWA2 are represented in Figures 6(a) and 6(b), respectively.

#### 2.2.4. Case 4: Simulated ECG Tracing with Phase-Reversal TWA

Arrhythmias can sometimes trigger a phase-reversal so that the alternans pattern changes from ABABAB to BABABA [5]. The simulated ECG tracing with phase-reversal TWA (PR_TWA) is designed to test the ability of the method in detecting phase-reversal TWA. PR_TWA tracing incorporates a stationary 10 *μV* TWA, which changes phase twice, at beats 40 and 80, respectively. This simulated case may also be used to help in the interpretation of realistic cases in which a beat is missed (false negative QRS detection) or wrongly inserted (false positive QRS detection). An example is represented in Figure 6(c).

**Figure 7:**
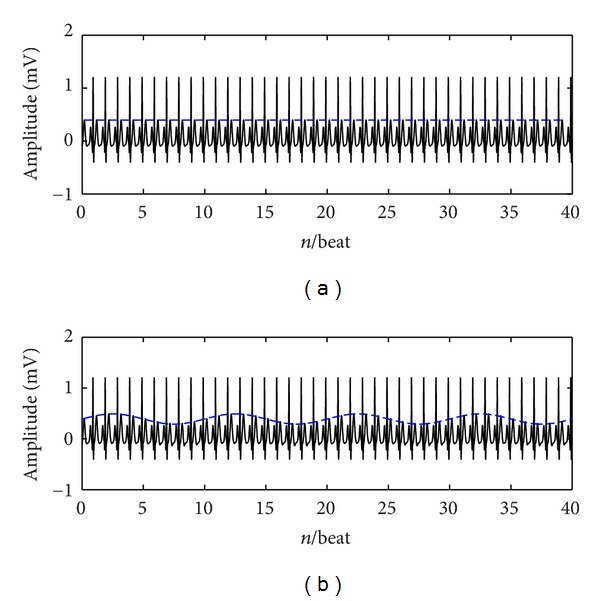
Two examples of the simulated tracings affected by baseline wanderings. (a) The simulated tracings without baseline; (b) the simulated tracings with 0.3 Hz baseline.

Finally the noise is also considered to be added to above simulated ECG tracings in this study. In clinical settings, power line interference is generally eliminated by a hardware filter. When computing the ACI indexes (2) the white noise is already taken into account. So baseline wandering is considered in the present simulated cases which might cause erroneous detection of TWA. Baseline wandering can be eliminated through the preprocessing state, but elimination related to T wave variability should be prevented because TWA is a specific case of it [4]. Based on these considerations, ECG simulations with baseline wandering are considered. Baseline wanderings are simulated with a sinusoid of 0.1 mV amplitude and various frequencies: 0.30, 0.71, and 1.50 Hz, respectively, which we denote as bw030, bw071, and bw150. These frequencies are, respectively, lower, equal, and greater than TWA frequency. The frequency of 0.30 Hz relates to a usual breathing pattern in patients. And the baseline fluctuations are simply added to each simulated ECG tracing. Two representative examples of our simulated ECG tracings, with and without baseline fluctuations, are displayed in Figure 7.

### 2.3. Clinical Cases

Two clinical data sets are considered in this study: ECG tracings from healthy subjects (H-subjects) and that from patients.

ECG tracings from H-subjects belong to the Digital Electrocardiology Study Databases of Liuhuaqiao Hospital, Guangzhou, which include 320 Holter ECG tracings from H-subjects. The study was approved by the institutional research ethics committee of Guangzhou Medical College, and it was conducted following the required rules for human subjects' research principles, according to the Declaration of Helsinki, as well as to Title 45, U.S. Code of Federal Regulations, Part 46, Protection of Human Subjects, Revised November 13, 2001, effective December 13, 2001. Each subject underwent 10-min ECG recording in resting conditions. Nine standard leads (V1–V6, I, II, and III) were recorded using equipment by Siemens-Elema AB and digitized at a sampling rate of 500 Hz with amplitude resolution of 0.6 *μ*V. Leads aVF, aVR, and aVL were derived from leads I, II, and III.

ECG tracings from patients belong to the T-Wave Alternans Challenge Database (TWACD) [17], which contains 100 multichannel ECG records sampled at 500 Hz with 16 bit resolution over a ±32 mV range. The subjects include patients with myocardial infarctions, transient ischemia, ventricular tachyarrhythmia, and other risk factors for sudden cardiac death, as well as healthy controls and synthetic cases with calibrated amounts of T-wave alternans. The databases are chosen for two reasons: one is that previous studies found T-wave alternans episodes, some of them related to annotated ischemic episodes. Another is that the databases are well-known and available by many research groups.

In the specific, a group of fourteen healthy subjects was compared with a group of fourteen patients. A subject was classified as belonging to the H-group when fulfilling the following criteria [18]:

1. no overt cardiovascular disease or history of cardiovascular disorders (including stroke, TIA, and peripheral vascular disease);
2. no history of high blood pressure (>150/90 mmHg);
3. not taking medication;
4. no other chronic illness (e.g., diabetes, asthma, chronic obstructive pulmonary disease, etc.);
5. diagnosed as being healthy if evaluated by a physician for cardiovascular-related syndrome (chest pain, palpitation, syncope);
6. normal physical examination;
7. sinus rhythm in 12-lead ECG without any suspicious abnormalities (e.g., signs of ventricular hypertrophy, inverted T-wave, intraventricular conduction disturbances);
8. normal echo and normal ECG exercise testing in the presence of suspicious ECG changes;
9. no pregnancy.

### 2.4. Statistics

To evaluate the ability of the presented method to quantify TWA, the other two related time-domain methods, which are the CM and the AMFM, are used here for comparison.

In our simulation study, the root mean square error (RMSE) in the estimate of TWA amplitudes is computed [13]:

(11) $$\begin{array}{c} \text{RMS}{\text{E}}_{M}=\sqrt{\frac{\sum_{n=1}^{N}{\left(\text{TW}{\text{A}}_{M}\left(n\right)-A\left(n\right)\right)}^{2}}{N}}, \end{array}$$

where *N* is the total number of beats in an ECG tracing, *A*(*n*) (relative to the *n*th beat) is assumed equal to the absolute value of the maximum difference between the *n*th and the (*n* + 1)th T-wave sample amplitude, and TWA_*M*_(*n*) is the estimated local TWA (relative to the *n*th beat) by the three competing methods. Subscript *M* is for either the HAM or the CM or the AMFM. In this study, the resolution of RMSE is  0.1 *μ*V, and the predefined *A*(*n*) are considered as constitutive reference TWA-amplitude signals (gold-standard).

When analyzing clinical data, the Lilliefors test was used to evaluate the hypothesis that estimated TWA had a normal distribution (significance was set at 5% level) over a population. Comparisons between normal distributions were performed using Student's *t-*test, whereas distributions that could not be considered normal would be compared using the Wilcoxon rank sum test. Statistical significant differences were assumed for *P* < 0.05.

## 3. Results

For the simulated data and clinical data set, ECG segments of 128 consecutive beats were randomly extracted and directly submitted to the AMFM, which does not require preprocessing [11]. Rather, a data preprocessing stage, described in Section 2.1, was performed prior to submitting the CM and the HAM.

### 3.1. Simulated Cases

For the simulated cases, results obtained from TWA analysis, by applying the CM, the AMFM, and the HAM, respectively, are reported in the tables below.

In Table 1 the results obtained from the simulated ECG tracing with no TWA (N_TWA) are reported. These three methods applying to tracings with no baseline yielded an accurate identification of TWA amplitude. In the presence of 0.30 and 1.50 Hz baseline wandering, a slight overestimation of TWA amplitude was produced by the CM. In the presence of baseline fluctuations with a frequency equal (0.71 Hz) to that of TWA, the strong overestimations of TWA amplitude were produced by the three methods.

In Table 2 the results obtained from the simulated ECG tracing with stationary TWA (S_TWA) are reported. In the presence of 0.30 and 1.50 Hz baseline wandering, the CM and the AMFM produced underestimation of TWA amplitude for different stationary TWA, while the HAM yielded an accurate identification of TWA (RMSE_HAM_ = 0 *μ*V). In the presence of baseline fluctuations at the TWA frequency of 0.71 Hz, the CM produced underestimation of TWA amplitude and the AMFM produced strong overestimation of TWA amplitude. While the HAM, produced a slight underestimation of TWA amplitude, showed a better ability to quantifying TWA amplitude in this case, and RMSE_HAM_ obtained  0 *μ*V for S_TWA10,  18.4 *μ*V for S_TWA50,  29.5 *μ*V for S_TWA100, respectively.

**Figure 8:**
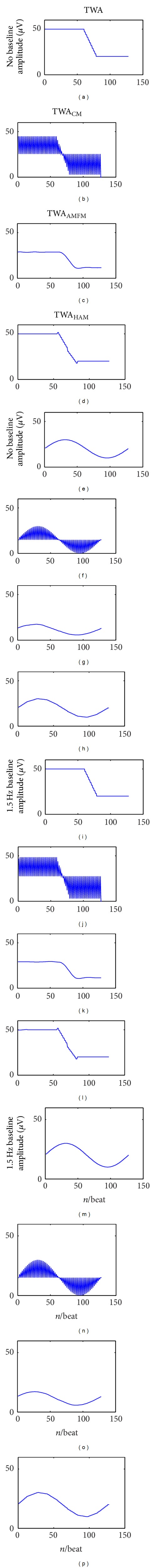
The time-varying TWA measurement results under different baseline conditions.

For TV_TWA1 and TV_TWA2 cases, the local TWA comparisons are considered because of time-varying amplitudes. A graphical representation of the results obtained from ECG simulations with the presence of time-varying TWA (TV_TWA1 and TV_TWA2) is depicted in Figure 8. The columns of panels from left to right display simulated TWA-amplitude signals (128 beats) and detected TWA-amplitude signals provided by the CM, the AMFM, and the HAM, respectively. For the cases of the simulated ECG tracing with 0.30 and 0.71 Hz baseline wandering, analogous results are obtained.

The root mean square errors obtained are reported in Table 3. The three methods were able to track the time course of TWA. But the local TWA-amplitude signals provided by the CM showed vigorous amplitude fluctuation, and RMSE_CM_ are higher then RMSE_AMFM_ and RMSE_HAM_ uniformly. The CM and the AMFM produced underestimation of TWA amplitude, which are the same as the above mentioned cases, while the HAM provided a good estimate of TWA (RMSE_HAM_ < 1.5 *μ*V, except the case of frequency of baseline equal to that of TWA).

In Table 4 the results obtained from the simulated ECG tracing with phase-reversal TWA (PR_TWA) are reported. The AMFM produced underestimation of TWA amplitude (40%) in the presence of 0.30 and 1.50 Hz baseline wandering, while the CM and the HAM produced good results (RMSE_CM_ = 0 *μ*V, RMSE_HAM_ = 0 *μ*V). In the presence of baseline fluctuations at the TWA frequency of 0.71 Hz, the three methods produced strong overestimation of TWA amplitude, but obviously the results provided by the HAM are more close to the simulated TWA (TWA_HAM_ = 19.7 *μ*V, RMSE_HAM_ = 13.1 *μ*V).

### 3.2. Clinical Cases

TWA levels quantified by the three competing methods in the H-subjects and patients data are reported in Table 5. The CM, the AMFM, and the HAM detected various levels of TWA in the same H-subjects and all patients. TWA was detected in two H-subjects by the CM and the AMFM, while only one H-subject was affected by TWA according to the HAM (Table 5). And the three methods detected the presence of TWA in all patients. TWA showed a normal distribution over patients' populations. Mean TWA values estimated by the HAM in H-subjects (0.5 ± 1.9 *μ*V) and patients (10.8 ± 3.7 *μ*V) were higher than the corresponding mean TWA estimates provided by the AMFM (H-subjects: 0.4 ± .91 *μ*V; patients: 8.6 ± 3.3 *μ*V) and the CM (H-subjects: 0.5 ± 1.3 *μ*V; patients: 9.5 ± 3.5 *μ*V). All these methods provided mean TWA estimates which showed significant differences between H-subject and patient groups.

The CM, the AMFM, and the HAM detected the presence of TWA in all patients and provided similar TWA estimates. The CM and the AMFM tend to underestimate TWA (Figure 8 and simulation study results), and this finding is confirmed by our clinical result.

## 4. Discussion

In this study four simulated cases were generated with characters of absence of TWA; presence of different kinds of stationary TWA; presence of two kinds of nonstationary (time-varying) TWA; and presence of phase-reversal TWA. The other two time-domain methods, namely, the CM and the AMFM, are compared with the HAM in TWA detection. Results of our simulation study indicate that the HAM allows detection and quantification of TWA better than the CM and the AMFM.

The CM was found to underestimate TWA amplitude in the simulated ECG tracing, since it assumed TWA being distributed along the entire length of the T-wave [19]. And in the case of ECG simulations with the presence of time-varying TWA, the CM produced the worst results compared with other methods (Figure 8 and Table 3).

The AMFM showed good performance of time-varying TWA detection, due to that its heart-rate adaptive-match-filter yielded the suppression of all ECG and interferences frequency components, while it produced strong overestimation of TWA amplitude in the presence of baseline fluctuations at the TWA frequency, and the reason and a potential solution were given in the literature [10].

We can find that the HAM yielded, in general, a more accurate TWA estimation in the simulated cases, although in the presence of baseline fluctuations with frequency equal to that of TWA the deviation from TWA amplitude was produced which are also produced by the CM, and the reason is that the accuracy of isoelectric line estimation by the cubic spline interpolation technique reduces. And all simulation cases showed that RMSE_HAM_ were systematically smaller than TWA_CM_ and TWA_AMFM_, even in the presence of baseline fluctuations at the TWA frequency of 0.71 Hz.

The HAM performs an amplitude corrections procedure based on the linear least squares fitting technique before calculating the local TWA, which further suppresses the interferences, and the local threshold criterion, integrated in the HAM, appears to help improve detecting accuracy. The limitation of the CM is that when computing the ACI, the exact location of the maximum amplitude difference between the two waves is lost, so that a mean (over T-wave) TWA amplitude value is provided (assumption of uniformly distributed TWA), while in our method TWA is measured by the maximum absolute value of the difference between the corrected matrixes for odd and even T-waves, which also improves the accuracy of TWA estimation. The baselines with various frequencies are considered in the simulated cases, and the test results also show that HAM is robust to the noise.

Our results relative to the clinical data highlighted consistency in the detection and quantification of TWA by the three different methods, and significant differences between H-subject and patient groups are manifested, as shown in Table 5, while the TWA amplitudes measured by the CM and the AMFM are slightly lower than that by the HAM. The results of our simulation test help interpreting the TWA data obtained from clinical cases.

## 5. Conclusions

A novel time-domain TWA detector is presented in this paper based on the correlation method and linear least squares fitting technique. Although the method is simple, it was validated using simulated ECG test signals with artificial TWA of various amplitudes and baseline wanderings and achieved good performance under reasonable levels of noise. The results of our simulation study indicate that the HAM provides a more accurate TWA estimation than the CM and the AMFM.

Results of TWA detection produced by the three methods in real clinical ECG records show high consistency, which confirms the TWA detection power of the hybrid method for clinical data, although the quantifying TWA amplitudes by the HAM are universally higher than that by the CM and the AMFM.

## Figures and Tables

**Table 1:** TWA amplitude measurements and errors evaluation for N_TWA case.

	TWA_CM_	TWA_AMFM_	TWA_HAM_	RMSE_CM_	RMSE_AMFM_	RMSE_HAM_
N_TWA(no bw)	0	0	0	0	0	0
N_TWA(bw030)	2	0	0	4.6	0	0
N_TWA(bw071)	34	200	23	34.4	385.9	22.6
N_TWA(bw150)	1	0	0	5.2	0	0

**Table 2:** TWA amplitude measurements and errors evaluation for S_TWA case.

	TWA_CM_	TWA_AMFM_	TWA_HAM_	RMSE_CM_	RMSE_AMFM_	RMSE_HAM_
S_TWA10(no bw)	6.5	5.7	10.0	3.2	4.23	0
S_TWA10(bw030)	6.5	5.8	10.0	4.7	4.2	0
S_TWA10(bw071)	12	219.7	10.1	21.6	209.7	0.2
S_TWA10(bw150)	7.2	5.9	10.0	2.6	4.2	0
S_TWA50(no bw)	36	28.6	50.0	14.3	21.4	0
S_TWA50(bw030)	36	28.8	50.0	14.8	28.9	0
S_TWA50(bw071)	17.3	198.8	32.0	32.4	198.8	18.4
S_TWA50(bw150)	38.2	29	50.0	11.7	29	0
S_TWA100(no bw)	74.9	57.7	100.0	25.3	42.3	0
S_TWA100(bw030)	74.9	57.5	70.0	25.6	42.4	0
S_TWA100(bw071)	54.7	176.2	100.0	45.0	76.0	29.5
S_TWA100(bw150)	79.2	57.6	100.0	21.1	42.4	0

**Table 3:** TWA errors evaluation for TV_TWA1 and TV_TWA2 cases.

	RMSE_CM_	RMSE_AMFM_	RMSE_HAM_
TV_TWA1(no bw)	13.8	12.7	0.9
TV_TWA1(bw030)	16.1	10.9	1.2
TV_TWA1(bw071)	25.5	296.9	14.9
TV_TWA1(bw150)	16.1	12.6	1.0
TV_TWA2(no bw)	6.9	2.8	0.5
TV_TWA2(bw030)	10.1	2.2	1.3
TV_TWA2(bw071)	19.9	342.1	15.1
TV_TWA2(bw150)	7.5	3.0	0.5

**Table 4:** TWA amplitude measurements and errors evaluation for PR_TWA cases.

	TWA_CM_	TWA_AMFM_	TWA_HAM_	RMSE_CM_	RMSE_AMFM_	RMSE_HAM_
PR_TWA(no bw)	10	6	10	0	2.8	0
PR_TWA(bw030)	10	6	10	5.1	3.1	1.3
PR_TWA(bw071)	32	230	19.7	22.5	348.3	13.1
PR_TWA(bw150)	10	6	10	0	3.2	0

**Table 5:** TWA amplitude measurements of clinical data applying the CM, the AMFM, and the HAM.

H-subjects	TWA_CM_	TWA_AMFM_	TWA_HAM_	TWACD	TWA_CM_	TWA_AMFM_	TWA_HAM_
1	0	0	0	TWA06	6.55	5.73	7.05
2	0	0	0	TWA09	6.91	6.21	8.11
3	0	0	0	TWA10	7.05	5.93	7.65
4	3.02	2.11	0	TWA18	3.75	3.23	4.25
5	0	0	0	TWA22	14.17	12.56	15.10
6	0	0	0	TWA23	12.91	10.83	13.47
7	0	0	0	TWA41	12.08	11.10	14.48
8	0	0	0	TWA46	5.95	4.91	7.55
9	4.12	4.08	6.11	TWA61	11.83	11.06	13.06
10	0	0	0	TWA71	7.12	7.10	8.80
11	0	0	0	TWA85	10.88	10.06	13.16
12	0	0	0	TWA92	13.10	12.55	14.21
13	0	0	0	TWA94	13.79	12.94	15.41
14	0	0	0	TWA99	7.16	6.38	8.23
	0.5 ± 1.3	0.4 ± 1.9	0.5 ± 1.3		9.5 ± 3.5*	8.6 ± 3.3*	10.8 ± 3.7*

*P < 0.05 when comparing H-subjects versus patients with the *t*-test for normal distributions.
